# PSMA Theranostics: Current Landscape and Future Outlook

**DOI:** 10.3390/cancers13164023

**Published:** 2021-08-10

**Authors:** Hanbo Zhang, Stella Koumna, Frédéric Pouliot, Jean-Mathieu Beauregard, Michael Kolinsky

**Affiliations:** 1 Department of Medical Oncology and Hematology, University of Manitoba, Winnipeg, MB R3E 0V9, Canada; hzhang2@cancercare.mb.ca; 2 Department of Diagnostic Imaging, Cross Cancer Institute, Edmonton, AB T6G 1Z2, Canada; Stella.Koumna@albertahealthservices.ca; 3 Department of Surgery, Université Laval, Québec City, QC G1R 3S1, Canada; frederic.pouliot@fmed.ulaval.ca; 4 Department of Radiology and Nuclear Medicine, Université Laval, Québec City, QC G1R 3S1, Canada; jean-mathieu.beauregard@crchudequebec.ulaval.ca; 5 Department of Medical Oncology, University of Alberta, Edmonton, AB T6G 1Z2, Canada

**Keywords:** prostate-specific membrane antigen, theranostics, positron emission tomography, radioligand therapy, prostate cancer

## Abstract

**Simple Summary:**

The prognosis for metastatic prostate cancer patients remains poor. Prostate-specific membrane antigen (PSMA) is overexpressed in prostate cancer and is a promising target for both imaging and therapy. In this review paper, we provide an overview of the evidence for PSMA-targeted imaging in prostate cancer, focusing on different imaging modalities and their theranostic applications. We will also review PSMA-targeted radioligand therapy, focusing on lutetium-177 radioligand therapy and alpha-emitting radioligand therapy with actinium-225. Combination regimens with lutetium-177 and other systemic therapy agents will be reviewed. Antibody-based radioimmunotherapy will also be discussed along with other noteworthy radionuclide agents.

**Abstract:**

Introduction: Prostate-specific membrane antigen (PSMA) is a promising novel molecular target for imaging diagnostics and therapeutics (theranostics). There has been a growing body of evidence supporting PSMA theranostics approaches in optimizing the management of prostate cancer and potentially altering its natural history. Methods: We utilized PubMed and Google Scholar for published studies, and clinicaltrials.gov for planned, ongoing, and completed clinical trials in PSMA theranostics as of June 2021. We presented evolving evidence for various PSMA-targeted radiopharmaceutical agents in the treatment paradigm for prostate cancer, as well as combination treatment strategies with other targeted therapy and immunotherapy. We highlighted the emerging evidence of PSMA and fluorodeoxyglucose (FDG) PET/CT as a predictive biomarker for PSMA radioligand therapy. We identified seven ongoing clinical trials in oligometastatic-directed therapy using PSMA PET imaging. We also presented a schematic overview of 17 key PSMA theranostic clinical trials throughout the various stages of prostate cancer. Conclusions: In this review, we presented the contemporary and future landscape of theranostic applications in prostate cancer with a focus on PSMA ligands. As PSMA theranostics will soon become the standard of care for the management of prostate cancer, we underscore the importance of integrating nuclear medicine physicians into the multidisciplinary team.

## 1. Introduction

Prostate cancer represents a significant public health problem in the world. It is the second most common cancer and fifth leading cause of cancer death among men in 2020 [1]. While most patients initially present with localized disease and can be offered curative intent therapies, including surgery and external-beam radiation therapy, a significant proportion of patients initially present with or will later develop metastatic disease. Androgen deprivation therapy (ADT) is the mainstay of therapy for metastatic and recurrent prostate cancer, but nearly all patients will eventually develop castration-resistance. Despite significant advances in systemic therapy options, the prognosis for metastatic castration-resistant prostate cancer (mCRPC) is poor, with overall survival under two years [2].

Prostate-specific membrane antigen (PSMA) is a type II transmembrane glycoprotein receptor that is overexpressed (up to 1000 times more than normal prostate cells) in most prostate cancers (>90%). PSMA is normally expressed in the renal tubules and duodenum, in which it plays an essential role in the processing and uptake of dietary folates, and in the brain, where it plays a major role in modulating the output of glutamate signaling. The biological function of PSMA in prostate cancer remains elusive, although evidence suggests its role in activation of PI3K-Akt signaling in prostate cancer through release of glutamate as messenger molecule [3]. The level of expression may increase with tumor dedifferentiation and castration resistance, although neuroendocrine prostate cancer may have a decreased level of PSMA [4]. Even though this antigen is not entirely specific to prostate cancer cells, it can serve as a target for imaging and therapy due to its tumoral overexpression with relatively low toxicity to healthy tissues showing uptake [5].

## 2. PSMA PET Imaging in Prostate Cancer

### 2.1. Imaging Modalities

Many PSMA-targeting small molecule and antibody agents have been developed and tested for imaging by single-photon emission computerized tomography (SPECT) and positron emission tomography (PET). Compared to anti-PSMA antibodies, small-molecule PSMA inhibitors are preferable as PET imaging agents due to faster tumor uptake and more rapid excretion, which enhance contrast and reduce radiation exposure [6,7]. Currently, the most developed radioligands for imaging include gallium-68-labeled PSMA-11 (^68^Ga-PSMA-11), gallium-68-labeled PSMA-I&T (^68^Ga-PSMA-I&T), and fluorine-18 (^18^F) labeled PSMA ligands, including ^18^F-DCFPyL and ^18^F-PSMA-1007. There is no consensus as to the optimal PSMA radioligand. Currently, ^68^Ga-PSMA-11 and ^18^F-DCFPyL are the only FDA-approved radioligands for PSMA-targeted PET imaging in men with prostate cancer [8,9].

The fluorinated agents have the advantage of a longer half-life which allows for central production and distribution over longer distances as well as lower positron energy that can lead to improved imaging quality. ^18^F-PSMA-1007 has been found to have diagnostic accuracy comparable to ^68^Ga-PSMA-11 for detection of biochemically recurrent prostate cancer, with the advantage of minimal excretion in the urinary tract, which allows for better detection of prostate bed recurrences [10,11]. However, cautions need to be taken when interpreting ^18^F-PSMA-1007 PET scans, as there have been reports of focal unspecific bone uptake, defined as focal mild-to-moderate uptake (SUVmax < 10.0) not obviously related to a benign or malignant cause [12].

### 2.2. Clinical Applications of PSMA-Targeted Imaging

PSMA-targeted imaging has been extensively explored in primary staging and restaging of prostate cancer. Conventional imaging with CT, MRI and bone scan have limited sensitivity and specificity for detecting occult metastatic disease, particularly in the setting of low PSA values [13]. Prospective studies have demonstrated advantage of PSMA-targeted imaging in both primary staging, as well as in the biochemical recurrence setting in providing useful clinical information that may ultimately change management [14,15,16,17]. Various international guidelines have recommended PSMA-PET imaging to be performed for the clarification of equivocal findings, particularly if the results will influence subsequent treatment decisions [18,19].

Recent evidence has suggested that treatment of the primary tumor or metastasis-directed therapy (MDT) in carefully selected patients with low-volume metastatic prostate cancer may confer a survival advantage [20]. Improved detection of metastatic prostate cancer using PSMA-targeted imaging may improve the success rate of MDT. ORIOLE is a randomized phase 2 study investigating whether stereotactic body radiation therapy (SBRT) for recurrent oligometastatic metastatic hormone-sensitive prostate cancer can improve survival outcomes compared to observation. Patients with oligometastatic disease (3 or fewer lesions) identified by conventional imaging were randomized to either SBRT to all metastatic lesions or observation. Salvage RT to the prostate bed or pelvis was permitted. Those randomized to MDT underwent ^18^F-DCFPyL PET/CT prior to MDT. The treating radiation oncologists were blinded to the result of PSMA scans and treatment plans were based on conventional imaging. The PSMA scans were compared to the SBRT treatment plans, and patients were categorized as having total or subtotal consolidation of PSMA-avid lesions. In the SBRT arm, disease progression at six months was 10% vs. 61% in the observation arm (*p* = 0.005). For those who received total consolidation, rate of new metastases at six months was 16% vs. 63% in those with subtotal consolidation (*p* = 0.006). Remarkably, median distant metastasis-free survival was 29 months in those with total consolidation vs. six months in those with any untreated lesions [21]. This study illustrates the important role of PSMA-imaging in guiding and optimizing therapeutic efficacy of MDT.

With the advent PSMA-targeted imaging, there are many ongoing clinical trials in oligometastatic-directed therapy (Table 1) in both the castration-sensitive and castration-resistant settings, involving various treatments strategies. Although it is encouraging to see early indications of benefit with the use of PSMA-targeted imaging in guiding treatment of oligometastatic prostate cancer, further evidence regarding efficacy, safety, and quality of life needs to be obtained prior to establishing this approach as the standard of care.

### 2.3. Biomarker for Evaluating Treatment Response

PSMA-targeted imaging has been explored as a biomarker for assessment of treatment response. However, this can be challenging as PSMA avidity can be affected by a multitude of factors such as castration-sensitive versus castration-resistant disease, timing and type of therapy received, and location of the lesions. PSMA expression appears to be upregulated with ADT and androgen-axis inhibitors such as enzalutamide in the first three months of treatment initiation [22,23]. PSMA response heterogeneity has also been reported for patients on ADT, with a higher PSMA response in nodal lesions compared to non-nodal lesions [24]. In one study, chemotherapy with docetaxel seems to have reasonable correlation between changes in PSMA avidity and RECIST measurements, although the correlations between PSA, RECIST and PSMA avidity were not completely concordant in those who were partial responders as per RECIST [25].

According to consensus statements on PSMA PET/CT response assessment criteria, from a panel of international experts recruited by European Association of Nuclear Medicine (EANM) and EAU (European Association of Urology), PSMA PET/CT should not be performed to assess response within three months after initiation of systemic therapy in hormone sensitive prostate cancer. The panel also recommended that PSMA PET/CT response assessment should be implemented and evaluated only in the context of clinical trials [26]. PSMA imaging as a response/progression biomarker is somewhat promising, although significant research in this field is required.

## 3. PSMA-Targeted Radioligand Therapy

### 3.1. Overview

**Figure 1:**
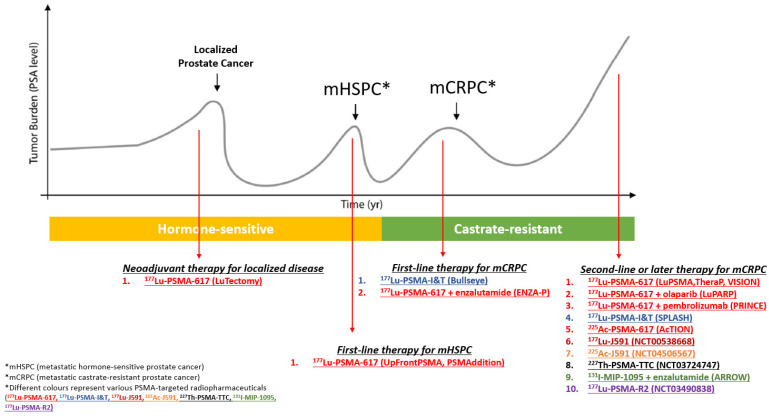
Schematic overview of selected PSMA theranostics clinical trials as of June 2021.

Prostate cancer is sensitive to radiotherapy (RT), in which various forms of RT, such as external beam RT (EBRT) and brachytherapy, have become standard treatment options for localized prostate cancer, and SBRT is currently explored in patients with oligometastatic prostate cancer. Systemic radiation with alpha particle-emitting calcium mimetic Radium-223 (^223^Ra) has been established for metastatic prostate cancer localized to the bone [27]. Concurrent with the development of PSMA-targeted imaging, the application of PSMA-targeted radioligand therapy is an ongoing area of great potential, and several PSMA-targeted radiopharmaceuticals have been developed for the treatment of advanced prostate cancer which enable the delivery of radiation to both bone and soft tissue tumor sites. Figure 1 illustrates a schematic overview of active PSMA-targeted radioligand therapy trials. Table 2 contains a descriptive summary of these trials.

### 3.2. PSMA and Fluorodeoxyglucose (FDG) PET/CT as Predictive Biomarker for PSMA Radioligand Therapy

The intensity of uptake (avidity) in PSMA PET/CT represents the PSMA expression in prostate cancer cells. The level of avidity generally increases with tumor dedifferentiation and castration resistance. However, dedifferentiated neuroendocrine prostate cancer maybe an exception in which PSMA-avidity can be low owing to suppression of PSMA gene (FOLH1) [4]. Pretreatment imaging is necessary prior to PSMA radioligand therapy to document the presence of PSMA-avid lesions. Currently, there is no established exact level of PSMA-avidity considered adequate for treatment eligibility. However, most centers consider 1.5 as the minimum ratio of the mean standardized uptake value (SUVmean) of the lesion to liver [28]. Based on a recent meta-analysis, PSMA-avidity is predictive of response to PSMA radioligand therapy with improved OS [29]. Higher PSMA-avidity is associated with higher radioligand uptake which allows cancer lesions to be exposed to a higher radiation dose [30]. Furthermore, the change in SUVmax of the metastatic lesions may have an association with PSA response [31].

^18^F-fluorodeoxyglucose (FDG) is a PET tracer that is preferentially taken up by cancer cells due to increased glucose metabolism known as “Warburg effect” [32]. FDG-PET is commonly used to stage, detect recurrences, and monitor response to therapies for various types of cancers such as lymphoma, melanoma, colorectal, esophageal, breast and lung [33]. In prostate cancer, the utility of FDG-PET is less established due to studies showing conflicting results for sensitivity [34,35]. This is likely due to the fact that prostate cancers demonstrate a wide variety of differentiation with varying degrees of aggressiveness. FDG uptake has been reported to be higher in prostate cancer with higher grades (Gleason score > 7) and more aggressive behaviors, especially in dedifferentiated and castration-resistant prostate cancer [35,36,37]. FDG-PET appears to be a prognostic biomarker in mCRPC patients. In a cohort study, 133 patients with mCRPC underwent imaging with ^18^F-FDG and ^18^F-fluorodihydrotestosterone (FDHT), which is an androgen receptor probe. Patients with more than 12 FDG-avid lesions had a significantly worse OS than their counterparts. The study also demonstrated an increased risk of death in patients with discordant lesions (positive FDG-avid lesion but negative androgen receptor expression with ^18^F-FDHT) [38].

Tumor sites with low PSMA-avidity and high FDG-avidity represent sites of aggressive disease which cannot be effectively targeted by radioligand therapy. Discordance between PSMA-PET and FDG-PET has been observed in very advanced mCRPC, with dismal OS of 2.5 months based on the LuPSMA trial [28]. Thus, FDG-PET maybe a useful imaging tool in conjunction with PSMA-PET to better select patients for PSMA radioligand therapy. This may help explain the higher response rates observed in the Australian trials of LuPSMA and TheraP in which stringent imaging selection criteria was applied which required patients to have highly PSMA avid disease without any FDG-positive/PSMA-negative lesions (discordant) [28,39]. Similarly, this patient selection strategy has been accepted and applied in peptide receptor radionuclide therapy (PRRT) with ^177^Lu-DOTATATE in patients with neuroendocrine tumors, in which FDG-PET is recommended to be used in conjunction with somatostatin-receptor (SSR) imaging to exclude patients with discordant lesions (FDG-positive/SSR-negative) from receiving PRRT treatment [40].

### 3.3. Lutetium-177 PSMA Radioligand Therapy

#### 3.3.1. Introduction

Lutetium-177 (^177^Lu) is a beta-emitting radioisotope that has a mean energy of 133.6 keV with maximum penetration depth of <2 mm and 6.7-days half-life [41,42]. Due to its favorable physical properties and the feasibility of post-treatment scintigraphy assessment, ^177^Lu labelled PSMA has been studied extensively to date and it has become a promising new therapeutic approach for treatment of mCRPC. The most studied of the ^177^Lu labelled PSMA radioligands include ^177^Lu-PSMA-I&T (imaging and therapy) and ^177^Lu-PSMA-617, with the latter being preferred owing to reduced kidney uptake [43]. Since 2015, an increasing number of studies have reported encouraging safety and efficacy of ^177^Lu-PSMA radioligand therapy [42]. According to a systemic review of 12 studies and 669 mCRPC patients who were treated with ^177^Lu-PSMA radioligand therapy in the 3rd line setting, 44% of treated patients had PSA decline ≥50% or more, with median overall survival of 14 months [43].

#### 3.3.2. ^177^Lu-PSMA-617

In the first prospective single-arm phase 2 study (LuPSMA), ^177^Lu-PSMA-617 has demonstrated PSA decline >50% in 57%, improvements in pain, and median PFS and OS of 7.6 months and 13.5 months, respectively in heavily pretreated mCRPC patients. The treatment was well tolerated with grade 1 xerostomia being the most common adverse event (87%) and grade 3–4 thrombocytopenia in 27% of patients [28].

Cabazitaxel is a chemotherapy agent for patients with mCRPC who have progressed after previous treatment with docetaxel. Based on the CARD study, cabazitaxel is the current standard of care for treatment for patients who have progressed on one of androgen-receptor-axis-targeted therapies (ARATs) (abiraterone or enzalutamide) and docetaxel [44]. TheraP is a randomized phase 2 clinical trial conducted by the Australian and New Zealand Urogenital and Prostate Cancer Trial Group (ANZUP) evaluating ^177^Lu-PSMA-617 (6.0–8.5 GBq intravenously every six weeks for up to six cycles) vs. cabazitaxel (20 mg/m^2^ intravenously every three weeks for up to 10 cycles) for mCRPC patients who have progressed on docetaxel. The majority (91%) of patients on the trial had prior ARAT. In this study, the ^177^Lu-PSMA-617 arm had an improved PSA response rate (PSA reduction ≥ 50%) of 66% vs. 37% for cabazitaxel, as well as improved one-year PFS rate of 19% vs. 3% (HR 0.63, 95% CI 0.46–0.86; *p* = 0.003) with a median follow-up of 18.4 months. Objective response rate (ORR) was significantly greater in ^177^Lu-PSMA-617 arm (49% vs. 24%, RR 2.1, 95%CI 1.1–4.1; *p* = 0.019). Pain response was also numerically higher with ^177^Lu-PSMA-617 (60% vs. 43% compared to chemotherapy. The safety profile was also favorable for ^177^Lu-PSMA-617, with Grade 3 or 4 AEs of 35% (neutropenia 4%, thrombocytopenia 11%, and fatigue 5%) versus 54% for cabazitaxel [39]. This study suggests that ^177^Lu-PSMA-617 is a promising alternative to cabazitaxel in men who have progressed following docetaxel for mCRPC.

The VISION study (NCT03511664) is an international phase 3 randomized trial of ^177^Lu-PSMA-617 (7.4 GBq every six weeks × six cycles) plus best standard/standard-of-care (SOC) versus best SOC care alone in heavily-pretreated mCRPC patients who have received at least one ARAT and were previously treated with 1–2 taxane chemotherapy regimens. The study enrolled 831 patients with 551 patients allocated to ^177^Lu-PSMA-617 + SOC. Over a median follow-up of 20.9 months, treatment with ^177^Lu-PSMA-617 significantly improved OS by a median of 4.0 months (median OS, 15.3 vs. 11.3 months; HR, 0.62 (95% CI: 0.52, 0.74); *p* < 0.001, one-sided), compared to SOC alone. The radiological PFS (rPFS) was also improved by treatment with ^177^Lu-PSMA-617 + SOC compared to SOC alone (median rPFS, 8.7 vs. 3.4 months; HR, 0.40 (99.2% CI: 0.29, 0.57); *p* < 0.001, one-sided). Overall ^177^Lu-PSMA-617 therapy was well tolerated. This is the largest and latest prospective study to date investigating ^177^Lu-PSMA-617 in treatment of mCRPC. The positive results of this study support the adoption of ^177^Lu-PSMA-617 as a standard armamentarium against advanced PSMA-positive mCRPC [45].

#### 3.3.3. ^177^Lu-PSMA-I&T

PSMA-I&T is a small molecule PSMA inhibitor initially developed in Germany in 2015. ^177^Lu-PSMA-I&T has similar PSMA-affinity, dosimetry and pharmacokinetics profile compared to ^177^Lu-PSMA-617 [44]. In a retrospective study, 56 patients with mCRPC received a total of 125 cycles, with mean dose of 5.76 GBq per cycle. PSA PFS was 13.7 months with 58.9% of patients achieving >50% PSA decline. The kidneys and parotid gland had the highest absorbed doses [46]. The largest cohort of patients treated with ^177^Lu-PSMA- I&T consisted of 100 mCRPC patients, who underwent 319 cycles with a mean dose of 7.4 GBq. PSA decline over 50% was seen in 38% of patients with PSA PFS and OS of 4.1 and 12.9 months, respectively. High grade hematological toxicities included 9% of anemia, 6% of neutropenia, and 4% of thrombocytopenia. High grade non-hematological toxicities were not observed [47].

SPLASH is a phase 3, open-label, randomized study evaluating the efficacy of ^177^Lu-PSMA-I&T (AKA. ^177^Lu-PNT2002) versus abiraterone or enzalutamide in delaying radiographic PFS in the second line setting after progression on 1st line ARAT (abiraterone or enzalutamide) in mCRPC patients. This study recently started recruitment in March 2021 with estimated enrollment of 415 participants (NCT04647526).

#### 3.3.4. Combination of ^177^Lu-PSMA-617 with ARATs

Preclinical observational studies have shown upregulation of PSMA messenger RNA production and PSMA receptor expression during AR inhibition [48]. In patients with CRPC, ARAT administration leads to increase in PSMA expression [23]. Furthermore, ARATs may lead to radiosensitization [49]. Based on these findings, it is postulated that combining ^177^Lu-PSMA radioligand therapy with ARAT may lead to improved tumor control in CRPC.

ENZA-P (NCT04419402) is a phase 2 randomized multicenter study evaluating enzalutamide in combination with ^177^Lu-PSMA-617 vs enzalutamide monotherapy as first-line therapy in patients with mCRPC who have sufficient PSMA expression on PSMA PET/CT. This study aims to recruit 160 men with mCRPC who are deemed at high-risk for early failure on enzalutamide monotherapy (two or more high-risk features as per PREVAIL and PROPHECY trials) [50,51]. Patients randomized to the treatment arm will receive up to four cycles of 7.5 GBq ^177^Lu-PSMA-617 in combination with 160 mg enzalutamide daily. Primary endpoint is PSA-PFS. Secondary endpoints include rPFS, OS, PSA-response rate, quality of life and adverse events. This study is expected to be completed in 2023.

PSMAddition (NCT04720157) is a phase 3 randomized study comparing ^177^Lu-PSMA-617 in combination with standard of care vs. standard of care in patients with metastatic hormone-sensitive prostate cancer (mHSPC). In this study, the standard of care is defined as a combination of ARAT and ADT. Approximately 1126 patients will be randomized in this study. This study is anticipated to start recruiting in May 2021.

Although there is a biological basis to support combining ARAT with ^177^Lu-PSMA-617, it will be important to determine long term safety and the impact of early use of ^177^Lu-PSMA-617 on subsequent lines of therapy, such as chemotherapy. Other important questions that need to be answered will include the consideration of optimal dose, schedule, and patient selection.

#### 3.3.5. Combination of ^177^Lu-PSMA-617 with DNA Damage Repair Inhibitors

Radiation induces single-stranded and double-stranded DNA breaks through formation of oxidative free radicals. The enzyme poly ADP ribose polymerase (PARP) is crucial in repairing radiation-induced single-stranded DNA breaks, which allows for radio-resistance of cancer cells [52]. Thus, PARP inhibition could lead to radiosensitization when combined with radioligand therapy. Furthermore, germline and somatic alterations in DNA damage repair (DDR) genes such as BRCA1/2 alterations are present in up to 12% and 25% of mCRPC, respectively [53,54]. Based on a study from Royal Marsden hospital, tumor tissues from 60 patients with mCRPC were analyzed for membranous PSMA expression (mPSMA) and DDR aberrations with next generation sequencing. This study found that DDR aberrations are associated with higher mPSMA expression [55]. PARP inhibitors such as olaparib and rucaparib have been shown to be effective against mCRPC with BRCA1/2 mutations and other DDR aberrations in prospective clinical trials [56,57,58]. As such, there is a strong biological rationale for combining DDR inhibitors with PSMA-targeted radioligand therapy in treatment of mCRPC.

Preclinical studies have shown synergistic anti-tumor effect of combination PARP inhibitor and radiation, including radioligand therapy. PARP inhibitors appear to be especially effective in enhancing radiation-induced tumor cell death at low doses [59,60]. LuPARP (NCT03874884) is an Australian phase 1 study examining the use of ^177^Lu-PSMA-617 with olaparib in men with mCRPC who have progressed on prior ARAT, and have PSMA-avid disease on imaging. ^177^Lu-PSMA-617 will be administered up to 6 cycles with the dose of olaparib escalated from 50 mg to 300 mg, in six increments. The primary endpoints are to establish dose limiting toxicities, maximum tolerated dose and recommended phase 2 dose. Secondary endpoints include safety and efficacy. Tissue and liquid biopsies (circulating tumor cells and DNA) will be collected and analyzed to identify predictive biomarkers of response and resistance.

Other DDR inhibitors that may have synergy with radioligand therapy include DNA-PK inhibitor AZD7648, ATM inhibitor AZD0156, and RNA polymerase I inhibitor CX-5461. Preclinical studies for these agents have shown synergy with radiation therapy [61,62,63].

#### 3.3.6. Combination of ^177^Lu-PSMA-617 with Immune Checkpoint Inhibitors

Anti-PD-1 monoclonal antibodies such as nivolumab and pembrolizumab, and anti-CTLA-4 monoclonal antibodies, such as ipilimumab, together referred to as immune-checkpoint inhibitors (ICI) are an important class of cancer therapies that have demonstrated significant activity, including some durable responses, in a number of cancers, including melanoma, non-small cell lung cancer, bladder cancer, and kidney cancer. These agents are now used as single agents or in combination with chemotherapies as first or second lines of treatment for approximately 50 cancer types with more than 3000 active clinical trials as of 2020 [64]. In prostate cancer, these agents have had disappointing results, suspected to be due to low levels of neo-antigens and immunogenic mutations seen in the majority of prostate tumors [65,66,67]. Research is currently focusing on identifying predictive biomarkers in prostate cancer, as well as strategies that will alter the tumor microenvironment which may lead to better response to ICIs. In particular, radiation is known to have a variety of immunomodulatory effects. It has been observed that following radiation of tumor sites can lead to shrinkage of other tumor sites that were not radiated. This phenomenon is termed “abscopal effect”, in which it is hypothesized that radiation induces immunogenic tumor cell death which leads to systemic antitumor immune response [68]. Various preclinical and clinical studies substantiated this hypothesis [69].

PRINCE (NCT03658447) is an Australian phase Ib/II study examining the combination of pembrolizumab with ^177^Lu-PSMA-617 in mCRPC patients who have progressed on ARAT. Patients with PSMA-avid disease are enrolled to receive pembrolizumab up to two years (35 cycles every three weeks) in combination with up to six cycles of ^177^Lu-PSMA-617 (six weeks apart). The primary endpoints include PSA response rate and safety. Secondary endpoints include rPFS, PSA-PFS, and OS. The study aims to enroll 37 patients with completion expected in October 2021.

#### 3.3.7. Other ^177^Lu PSMA Radioligand Therapy Trials in Prostate Cancer

Clinical trials are underway to examine the role of ^177^Lutetium PSMA radioligand therapy earlier in the prostate cancer natural history. The LuTectomy trial (NCT04430192) is an Australian open label, phase I/II non-randomized study evaluating neoadjuvant ^177^Lu-PSMA-617 (one or two cycles) followed by radical prostatectomy + pelvic lymph node dissection in patients with high risk localized or locoregional advanced prostate cancer and PSMA-avid disease. Accrual for this study has begun with estimated primary completion date in August of 2022.

The Bullseye trial (NCT04443062) is a Dutch randomized, open label, multi-center, phase 2 study testing ^177^Lu-PSMA-I&T in oligometastatic HSPC. Patients with PSMA-avid disease with five or less metastatic lesions are randomized to either two cycles of ^177^Lu-PSMA-I&T given six weeks apart vs. standard of care. The estimated enrollment size is 58 patients with estimated primary completion in January 2023. The important clinical rationale of this study is that many patients with oligometastatic prostate cancer do not qualify for local MDT treatment such as surgery or EBRT due to tumor location or prior therapy, in which targeted radioligand therapy for these lesions maybe beneficial.

UpFrontPSMA (NCT04343885) is an Australian phase 2 randomized clinical trial comparing the efficacy of two cycles ^177^Lu-PSMA-617 given every six weeks followed by docetaxel chemotherapy every three weeks for six cycles, versus docetaxel chemotherapy on its own in patients with newly-diagnosed high volume mHSPC, defined as PSMA-avid visceral metastases, or ≥4 PSMA-avid bone lesions with one or more outside the vertebral column and pelvis as seen on ^68^Ga-PSMA PET/CT. The study aims to enroll 140 patients with estimated primary completion in April of 2024.

### 3.4. Other Notable Beta-Emitting PSMA-Targeted Radionuclide Therapy Agents

#### 3.4.1. ^131^I-MIP-1095

Iodine-131(^131^I)-MIP-1095 is a PSMA-targeted small-molecule inhibitor radionuclide therapy developed by Molecular Insight Pharmaceuticals, Inc. It was one of the first agents used in radiopharmaceutical therapy against prostate cancer. ^131^I is a beta-emitter having a similar particle range and half-life as ^177^Lu (eight vs. 6.7 days, respectively), but with much more abundant gamma emission, which makes it less ideal from toxicity and radiation safety perspectives [70].

A study testing a single cycle of ^131^I-MIP-1095 in 28 mCRPC patients showed PSA decrease of >50% in 60.7% of patients with median time to PSA progression of 126 days. Seven of the 28 patients experienced transient xerostomia with all reported symptoms resolved within 3–4 weeks. Although hematological toxicities were infrequent (two patients with grade 3 thrombocytopenia, one patient was grade 3 leukopenia), thrombocytopenia lasted multiple months [70]. In a subsequent study involving 36 mCRPC patients, 70.6% experienced PSA decrease >50% with median PSA progression of 116 days. Patients received an additional dose at PSA progression. However, only 65.2% experienced any PSA decline with only much higher percentage grade 3 thrombocytopenia of 13%, compared to 5.9% after first dose. Grade 3 xerostomia was observed in 13%, which was not seen after the first dose [71]. ARROW (NCT03939689) is a randomized, multicenter, controlled phase 2 study is currently underway to evaluate the safety and efficacy of ^131^I-MIP-1095 in combination with enzalutamide compared to enzalutamide alone in patients with PSMA-avid mCRPC who have progressed on abiraterone.

#### 3.4.2. ^177^Lu-PSMA-R2

^177^Lu-PSMA-R2 is being developed by Advanced Accelerator Applications (a subsidiary of Novartis). PSMA-R2 is a urea-based PSMA-targeting small molecule inhibitor. In preclinical studies, ^177^Lu-PSMA-R2 has rapid and specific uptake in mice bearing prostate cancer tumors, with rapid elimination through urinary system [72]. A phase 1/2 dose escalation study of ^177^Lu-PSMA-R2 in patients with PSMA-avid mCRPC is currently being conducted, with planned completion in June 2022 (NCT03490838).

### 3.5. Alpha-Emitting PSMA-617 Radioligand Therapy

#### 3.5.1. Introduction

Alpha particle radiation is characterized by a significantly higher linear energy transfer (LET) compared to beta particle radiation (50–230 keV/μm vs. 0.2 keV/μm), which allows for a greater chance of inducing unrepairable DNA double-strand breaks in cancer cell with increased cytotoxic potential. Furthermore, alpha particles have a shorter path in tissue (50–100 μm) compared with beta articles (1000–10,000 μm) [73,74]. The short range of alpha particles has the potential of minimizing cytotoxic damage in non-targeted cells, potentially leading to less hematological toxicity, especially in patients with wide-spread bone metastases with diffuse bone marrow infiltration [75]. This is exemplified by ^223^Ra, which is a first-in-class alpha particle-emitting agent approved for mCRPC with symptomatic bone metastases [27]. However, ^223^Ra treatment in mCRPC is limited by the fact that it is a bone-seeking agent that does not target soft-tissue metastases, and even within bone metastases the tumor cells are only reached via cross-fire radiation. The discovery of PSMA allowed for the delivery of alpha-emitting radionuclides more selectively to the tumor.

#### 3.5.2. ^225^Ac-PSMA-617

Actinium-225 (^225^Ac)-PSMA-617 has been the most studied PSMA-targeting alpha particle therapy. Based on a preliminary report from a single-center study, ^225^Ac-PSMA-617 (100 kBq/kg every two weeks) resulted in complete PSA and imaging response in two patients who received greater than eight prior systemic therapies [76]. In the full report including 14 patients, PSA response was seen in 75% of patients. All-grade hematological AEs occurred in six patients and xerostomia occurred in eight patients. Xerostomia was the dose-limiting toxicity with 100 kBq/kg considered the maximum tolerable dose [77]. ^225^Ac-PSMA-617 is also active in mCRPC patients who have progressed after ^177^Lu-PSMA-617. In a group of 26 heavily pre-treated (median of six prior lines of systemic therapy) mCRPC patients who have progressed on ^177^Lu-PSMA-617, all of them received ^225^Ac-PSMA-617 under a compassionate use program in Germany. This study reported substantial PSA decline of >50% in 65% of patients with PFS and OS of 4.1 and 7.7 months, respectively. High grade hematological toxicities were anemia (35%), leucopenia (27%), and thrombocytopenia (19%). All patients experienced xerostomia, which led to treatment discontinuation in about a quarter of patients [78].

^225^Ac-PSMA-617 has also been reported to have good activity against CNS metastases. Although rare in prostate cancer, brain metastases tend to occur in late stage CRPC and are associated with very poor survival. Treatment is generally limited to surgical resection or EBRT. In a case report, ^225^Ac-PSMA-617 achieved complete molecular imaging response on repeat ^68^Ga-PSMA PET in an mCRPC patient with PSMA-avid cerebral metastases after just 1 cycle of therapy. The patient had near complete PSA response after two cycles (788.63 μg/L to 0.32 μg/L [79]. This case highlights the potential of ^225^Ac-PSMA-617 being an effective treatment for brain metastases as PSMA-617 crosses the blood–brain barrier. However, no similar report has been seen yet with ^177^Lu-PSMA-617.

AcTION (NCT04597411) is a prospective phase 1, open-label, international, dose escalation study to evaluate the safety of ^225^Ac-PSMA-617 in men with PSMA-avid mCRPC. The study has started in April of 2021. This study will hopefully help to address how best to minimize xerostomia while still achieving an adequate response.

#### 3.5.3. ^213^Bi-PSMA-617

Bismuth-213 (^213^Bi) is a mixed alpha and beta emitter with a short half-life of 45.6 min. It demonstrated pre-clinical activity when tagged to PSMA-targeting antibody J591 [80,81]. Thus far, there has been only one clinical case report of ^213^Bi-PSMA-617 in which one patient with mCRPC was treated with two cycles. This patient achieved PSMA-imaging response and biochemical response with decrease in PSA level from 237 μg/L to 43 μg/L [82]. Due to its short half-life, it is logistically challenging for therapeutic use. It also has an inferior therapeutic index compared to ^225^Ac-PSMA-617 based on a dosimetry study [83]. Currently, this PSMA radioligand therapy agent has not been explored much further in its clinical application against prostate cancer.

## 4. Anti-PSMA Radioimmunotherapy

### 4.1. Lutetium-177-J591 Antibody

The extracellular domain of PSMA also makes an accessible target for antibodies. Anti-PSMA radioimmunotherapy actually predates the development of small molecule-based radioligand therapy. A notable example is J591 antibody, which was initially characterized in 1997, and it has been the most explored antibody for PSMA-targeted radioimmunotherapy to date [84]. ^177^Lu-J591 was initially tested in a phase I study in 2005 in 35 mCRPC patients who received up to three doses. Myelosuppression was dose limiting and the maximum-tolerated dose was 70 mCi/m^2^. Biological activity was seen with four patients experiencing >50% declines in PSA lasting up to eight months with 46% of patients have PSA stabilization for a median of 60 days [85]. In a phase 2 study investigation single doses of ^177^Lu-J591, median OS was 21.8 months in mCRPC patients who received a single dose of 70 mCi/m^2^, compared to 11.9 months in those who received 65 mCi/m^2^. However, higher dose led to higher grade 4 thrombocytopenia and neutropenia (53% vs. 27%, and 48% vs. 0%, respectively) [86].

Dose-fractionation of ^177^Lu-J591 appears to be a promising strategy to increase cumulative radiation in order to improve anti-tumor activity while mitigating hematological toxicity. In a phase 1/2 study, 49 mCRPC patients received fractionated doses of ^177^Lu-J591 ranging from 20 to 45 mCi/m^2^ × 2 two weeks apart. The recommended phase 2 doses were 40 mCi/m^2^ and 45 mCi/m^2^ × 2. At the higher dose, median OS was 42.3 months vs. 19.6 months in patients who received lower dose. The higher dose led to higher grade 4 thrombocytopenia and neutropenia than lower dose (58.8% vs. 43.8%, and 35.3% vs. 31.3%, respectively), with no high-grade hemorrhagic episodes in any of the patients [87].

### 4.2. Actinium-225-J591 Antibody

The cause of higher hematological toxicity for ^177^Lu-J591 is likely attributed to monoclonal antibodies being large molecules with slower clearance from the circulation compared to small molecules such as PSMA-617 [84]. However, compared to PSMA-617, J591 does not demonstrate uptake in the salivary gland or kidneys [7]. Based on this feature, it is hypothesized that ^225^Ac-J591 could mitigate the xerostomia and nephrotoxicity seen in ^225^Ac-PSMA-617. In a phase 1 trial, 22 mCRPC heavily-pretreated patients were treated with seven dose levels of ^225^Ac-J591. The treatment was very well tolerated and the MTD was not reached. Only one patient treated with 80 KBq/kg had grade 4 anemia and thrombocytopenia. There were no DLT reported in 6 patients that were treated at the highest planned dose of 93.3 KBq/kg. Xerostomia was only seen in patients with prior ^177^Lu-PSMA-617 therapy. PSA decline was seen in 64% of patients with 41% of patients experienced ≥ 50% PSA decline. Enrollment in the expansion cohort is currently being completed [88]. Given the above-mentioned success of the dose-fractionation strategy with ^177^Lu-J591, a phase I/II dose-escalation study of fractionated and multiple dose ^225^Ac-J591 is currently ongoing for mCRPC patients (NCT04506567).

### 4.3. Thorium-227-PSMA-TTC Antibody

Thorium-227 (^227^Th) is an alpha-particle emitter with a long half-life of 18.7 days, which allows for ease of transportation and preparation of the radiopharmaceutical. ^227^Th can be efficiently complexed with octadenate 3, 2-hydroxypryridinone (3,2-HOPO) chelator convalently linked to antibodies at ambient room temperature. ^227^Th-PSMA-TTC is a novel, fully human antibody attached to ^227^Th, which has recently been developed by Bayer and has demonstrated very strong antitumor efficacy in animal models with prostate cancer [89]. Currently, it is being tested in a large multi-center, international, phase 1, open-label study in mCRPC pre-treated mCRPC patients. The study is active with a planned enrollment of 157 participants and estimated completion in July 2024 (NCT03724747).

## 5. PSMA-Targeted Bispecific T-Cell Engager (BiTE) Immunotherapy

BiTE (bispecific T-cell engager) therapy is a novel targeted immunotherapy approach that engages the patient’s own T cells to kill cancer cells. The BiTE molecule binds to a tumour-associated antigen on targeted cells and CD3 on T cells which leads to T-cell proliferation, cytokine production, and T-cell-mediated killing of cancer cells. The advantage of this treatment approach over conventional ICI is such that it can bypass the conventional pathway of T-cell receptor activation and may be active, independent of the tumour’s genetic background [90]. BiTE therapy has shown great efficacy for treatment of certain hematological cancers [91,92]. The emergence of this novel therapy has renewed hope for immunotherapy in prostate cancer, where efficacy for ICI have been disappointing, owing to the suboptimal immunogenic microenvironment seen in prostate tumours [65,66,67].

Pasotuxizumab (AMG 212) is a 55kDa BiTE molecule that binds to CD3 on T cells and also PSMA on prostate cancer cells, thereby activating the patient’s own T cells to eliminate PSMA-expressing prostate cancer cells. Preclinical studies have demonstrated delayed tumour growth and shrinkage in human cancer xenograft models [91]. A phase I dose-escalation study of once daily subcutaneous (SC) pasotuxizumab in patients with multi-treated mCRPC demonstrated remarkable safety, with only 16% drug-related AEs reported. The SC maximum tolerated dose was 172 μg/d. PSA responders (>50% PSA decline) occurred in 29%, including two long-term responders. One such patient achieved near-complete radiological response, having received six prior therapies including 177Lu-PSMA-617 prior to commencing with pasotuxizumab, and then had 500 days to tumor progression [93]. Despite the encouraging results, further studies are required to fully elucidate the role of BiTE therapy in the treatment of mCRPC. Whether this treatment only works for PSMA-avid disease, as well as the exact specificity for PSMA, remain to be addressed.

## 6. Conclusions

Based on extensive research conducted thus far, PSMA theranostics represents a rapidly emerging strategy in the management of prostate cancer. PSMA-targeted imaging has demonstrated excellent sensitivity and specificity for the detection of prostate cancer compared to conventional imaging, with preliminary evidence demonstrating that the result impacts clinical practice. There are many potential clinical applications of PSMA-targeted imaging, with the most encouraging being the earlier detection of disease, leading to earlier therapeutic interventions. However, the impact on survival of an earlier initiation of therapy based on PSMA-targeted imaging is not yet apparent. Furthermore, PSMA-targeted radioligand therapy is a highly promising way to treat advanced prostate cancer, with recent evidence of rPFS and OS benefits. More research is underway to validate efficacy as well as improve safety and outcomes at earlier stages of the disease. Future research directions include the development of standardized methods for patient selection, response assessment, cost–benefit analysis, and the optimization of production and supply of radiopharmaceuticals. Considering that PSMA theranostics will soon become a standard of care in the management of prostate cancer, it will be crucial to integrate nuclear medicine physicians into the multidisciplinary team alongside medical oncologists, radiation oncologists, urologists, and radiologists in order to optimize the care of patients with this complex malignancy.

## Figures and Tables

**Table 1:** Ongoing clinical trials in oligometastatic-directed therapy using PSMA PET imaging.

Clinical Trial Identifier.	Study Description	Phase
NCT04302454	MDT +/− ADT in oligo-recurrent hormone-sensitive prostate cancer (ADOPT)	3
NCT04222634	MDT in oligometastatic mCRPC (MEDCARE)	2
NCT03902951	SBRT with ADT, abiraterone and apalutamide for oligometastatic hormone-sensitive prostate cancer	2
NCT03795207	SBRT +/− durvalumab in oligometastatic recurrent hormone-sensitive prostate cancer (POSTCARD)	2
NCT03569241	MDT +/− whole pelvic radiation for oligorecurrent nodal prostate cancer (STORM)	2
NCT03298087	Newly diagnosed oligometastatic hormone-sensitive prostate cancer treated with radical prostatectomy, metastasis-direct SBRT, ADT, abiraterone and apalutamide	2
NCT02974075	Salvage lymph node dissection of nodal recurrence after radical prostatectomy with curative intent	1/2

**Table 2:** Selected key PSMA theranostics clinical trials as of June 2021.

Clinical Trial Identifier	Brief Study Description	Phase
NCT04430192	^177^Lu-PSMA-617 prior to prostatectomy (LuTectomy)	1/2
NCT04343885	Sequential ^177^Lu-PSMA-617 + docetaxel vs. docetaxel in mHSPC (UpFrontPSMA)	2
NCT04720157	^177^Lu-PSMA-617 + SOC vs. SOC alone in mHSPC (PSMAddition),	3
NCT04443062	^177^Lu-PSMA-617 in oligometastatic mHSPC (Bullseye)	2
NCT04419402	Enzalutamide + ^177^Lu-PSMA-617 vs. Enzalutamide alone in mCRPC (ENZA-P))	2
ACTRN12615000912583	^177^Lu-PSMA-617 in progressive mCRPC (LuPSMA)	2
NCT0392428	^177^Lu-PSMA-617 vs. cabazitaxel in progressive mCRPC (TheraP)	2
NCT03511664	^177^Lu-PSMA-617 + SOC vs. SOC in progressive mCRPC (VISION)	3
NCT03874884	^177^Lu-PSMA-617 + olaparib in progressive mCRPC (LuPARP)	1
NCT03658447	^177^Lu-PSMA-617 + pembrolizumab in progressive mCRPC (PRINCE)	1/2
NCT04647526	^177^Lu-PSMA-I&T vs. ARAT in progressive mCRPC (SPLASH)	3
NCT04597411	^225^Ac-PSMA-617 in progressive mCRPC (AcTION)	1
NCT00538668	^177^Lu-J591 Antibody in progressive mCRPC	1
NCT04506567	^225^Ac-J591 Antibody in progressive mCRPC	1/2
NCT03724747	^227^Th-PSMA-TTC in progressive mCRPC	1
NCT03939689	^131^I-MIP-1095 + enzalutamide in progressive mCRPC (ARROW)	1
NCT03490838	177Lu-PSMA-R2 in progressive mCRPC	1/2

## References

[1] Sung H., Ferlay J., Siegel R.L., Laversanne M., Soerjomataram I., Jemal A., Bray F. (2020). Global cancer statistics 2020: GLOBOCAN estimates of incidence and mortality worldwide for 36 cancers in 185 countries. CA Cancer J. Clin..

[2] Kirby M., Hirst C., Crawford E.D. (2011). Characterising the castration-resistant prostate cancer population: A systematic review. Int. J. Clin. Pract..

[3] Kaittanis C., Andreou C., Hieronymus H., Mao N., Foss C.A., Eiber M., Weirich G., Panchal P., Gopalan A., Zurita J. (2018). Prostate-specific membrane antigen cleavage of vitamin B9 stimulates oncogenic signaling through metabotropic glutamate receptors. J. Exp. Med..

[4] Bakht M., Derecichei I., Li Y., Ferraiuolo R.M., Dunning M., Oh S.W., Hussein A., Youn H., Stringer K.F., Jeong C.W. (2018). Neuroendocrine differentiation of prostate cancer leads to PSMA suppression. Endocr. Relat. Cancer.

[5] Sokoloff R.L., Norton K.C., Gasior C.L., Marker K.M., Grauer L.S. (2000). A dual monoclonal sandwich assay for prostate-specific membrane antigen: Levels in tissues, seminal fluid and urine. Prostate.

[6] Pandit-Taskar N., O’Donoghue J.A., Beylergil V., Lyashchenko S., Ruan S., Solomon S.B., Durack J.C., Carrasquillo J.A., Lefkowitz R.A., Gonen M. (2014). ^89^Zr-huJ591 immuno-PET imaging in patients with advanced metastatic prostate cancer. Eur. J. Nucl. Med. Mol. Imaging.

[7] Pandit-Taskar N., O’Donoghue J.A., Durack J.C., Lyashchenko S.K., Cheal S.M., Beylergil V., Lefkowitz R.A., Carrasquillo J.A., Martinez D.F., Fung A.M. (2015). A phase I/II study for analytic validation of ^89^Zr-J591 immunoPET as a molecular imaging agent for metastatic prostate cancer. Clin. Cancer Res..

[8] FDA Approves Gallium-68 PSMA-11 for PSMA-Targeted PET Imaging in Prostate Cancer. https://ascopost.com/news/december-2020/fda-approves-gallium-68-psma-11-for-psma-targeted-pet-imaging-in-prostate-cancer/?bc_md5=7b5c61365d6220a9cca0c0ba65753923&utm_source=TAP%2DEN%2D120120&utm_medium=email.

[9] FDA Approves PSMA PET Imaging Agent ^18^F-DCFPyL for Prostate Cancer. http://https://www.urologytimes.com/view/fda-approves-psma-pet-imaging-agent-18f-dcfpyl-for-prostate-cancer.

[10] Rahbar K., Weckesser M., Ahmadzadehfar H., Schäfers M., Stegger L., Bögemann M. (2018). Advantage of ^18^F-PSMA-1007 over ^68^Ga-PSMA-11 PET imaging for differentiation of local recurrence vs. urinary tracer excretion. Eur. J. Nucl. Med. Mol. Imaging.

[11] Giesel F.L., Knorr K., Spohn F., Will L., Maurer T., Flechsig P., Neels O., Schiller K., Amaral H., Weber W.A. (2019). Detection efficacy of ^18^F-PSMA-1007 PET/CT in 251 patients with biochemical recurrence of prostate cancer after radical prostatectomy. J. Nucl. Med..

[12] Grünig H., Maurer A., Thali Y., Kovacs Z., Strobel K., Burger I.A., Müller J. (2021). Focal unspecific bone uptake on ^18^F-PSMA-1007 PET: A multicenter retrospective evaluation of the distribution, frequency, and quantitative parameters of a potential pitfall in prostate cancer imaging. Eur. J. Nucl. Med. Mol. Imaging.

[13] Hövels A.M., Heesakkers R.A., Adang E.M., Jager G.J., Strum S., Hoogeveen Y.L., Severens J.L., Barentsz J.O. (2008). The diagnostic accuracy of CT andMRI in the staging of pelvic lymph nodes in patients with prostate cancer: A metaanalysis. Clin. Radiol..

[14] Pienta K.J., Gorin M.A., Rowe S.P., Carroll P.R., Pouliot F., Probst S., Saperstein L., Preston M.A., Alva A.S., Patnaik A. (2021). A phase 2/3 prospectiev multicenter study of the diagnostic accuracy of prostate-specific membrane antigen PET/CT with ^18^F-DCFPyL in prostate cancer patients (OSPREY). J. Urol..

[15] Hofman M.S., Lawrentschuk N., Francis R.J., Tang C., Vela I., Thomas P., Rutherford N., Martin J.M., Frydenberg M., Shakher R. (2020). proPSMA Study Group Collaborators. Prostate-specific membrane antigen PET-CT in patients with high-risk prostate cancer before curative-intent surgery or radiotherapy (proPSMA): A prospective, randomised, multicentre study. Lancet.

[16] Calais J., Fendler W.P., Eiber M., Gartmann J., Chu F., Nickols N.G., Reiter R.E., Rettig M.B., Marks L.S., Ahlering T.E. (2018). Impact of ^68^Ga-PSMA-11 PET/CT on the management of prostate cancer patients with biochemical recurrence. J. Nucl. Med..

[17] Morris M.J., Carroll P.R., Saperstein L., Pouliot F., Josephson D., Wong J.Y.C., Pantel A.R., Cho S.Y., Gage K., Piert M. (2020). Impact of PSMA-targeted imaging with ^18^F-DCFPyL-PET/CT on clinical management of patients (pts) with biochemically recurrent (BCR) prostate cancer (PCa): Results from a phase III, prospective, multicenter study (CONDOR). J. Clin. Oncol..

[18] Trabulsi E.J., Rumble R.B., Jadvar H., Hope T., Pomper M., Turkbey B., Rosenkrantz A.B., Verma S., Margolis D.J., Froemming A. (2020). Optimum Imaging Strategies for Advanced Prostate Cancer: ASCO Guideline. J. Clin. Oncol. Off. J. Am. Soc. Clin. Oncol..

[19] Mottet N., van den Bergh R.C.N., Briers E., Van den Broeck T., Cumberbatch M.G., Santis M.D., Fanti S., Fossati N., Gandaglia G., Gillessen S. (2020). EAU-EANM-ESTRO-ESUR-SIOG Guidelines on Prostate Cancer-2020 Update. Part 1: Screening, Diagnosis, and Local Treatment with Curative Intent. Eur. Urol..

[20] Broughman J.R., Fleming C.W., Mian O.Y., Stephans K.L., Tendulkar R.D. (2020). Management of Oligometastatic Prostate Cancer. Appl. Radiat. Oncol..

[21] Phillips R., Shi W.Y., Deek M., Radwan N., Lim S.J., Antonarakis R.S., Rowe S.P., Ross A.R., Gorin M.A., Deville C. (2020). Outcomes of observation vs stereotactic ablative radiation for oligometastatic prostate cancer: The ORIOLE phase 2 randomized clinical trial. JAMA Oncol..

[22] Aggarwal R., Wei X., Kim W., Small E.J., Ryan C.J., Carroll P., Cooperberg M., Evans M.J., Hope T. (2018). Heterogeneous flare in prostate-specific membrane antigen positron emission tomography tracer uptake with initiation of androgen pathway blockade in metastatic prostate cancer. Eur. Urol. Oncol..

[23] Emmett L., Yin C., Crumbaker M., Hruby G., Kneebone A., Epstein R., Nguyen Q., Hickey A., Ihsheish N., O’Neill G. (2019). Rapid modulation of PSMA expression by androgen deprivation: Serial ^68^Ga PSMA-11 PET in men with hormone sensitive and castrate resistant prostate cancer commencing androgen blockade. J. Nucl. Med. Epub..

[24] Murthy V., Gupta P., Agrawal A., Maitre M., Krishnatry R., Rangarajan V. (2017). Ga-68 PETCT response to androgen deprivation therapy in patients with prostate cancer. Eur. Urol. Suppl..

[25] Seitz A.K., Rauscher I., Haller B., Kronke M., Luther S., Heck M.M., Maurer T. (2018). Preliminary results on response assessment using ^68^Ga-HBED-CC-PSMA PET/CT in patients with metastatic prostate cancer undergoing docetaxel chemotherapy. Eur. J. Nucl. Med. Mol. Imaging.

[26] Fanti S., Goffin K., Hadaschik B.A., Herrmann K., Maurer T., MacLennan S., Oprea-Lager D.E., Oyen W.J., Rouvière O., Mottet N. (2021). Consensus statements on PSMA PET/CT response assessment criteria in prostate cancer. Eur. J. Nucl. Med. Mol. Imaging.

[27] Parker C., Nilsson S., Heinrich D., Helle S.I., O’Sullivan J.M., Fosså S.D., Chodacki A., Wiechno P., Logue J., Seke M. (2013). Alpha emitter radium-223 and survival in metastatic prostate cancer. N. Engl. J. Med..

[28] Hofman M.S., Violet J., Hicks R.J., Ferdinandus J., Thang S.P., Akhurst T., Iravani A., Kong G., Kumar A.R., Murphy D.G. (2018). [^177^Lu]-PSMA-617 radionuclide treatment in patients with metastatic castration-resistant prostate cancer (LuPSMA trial): A single-centre, single-arm, phase 2 study. Lancet Oncol..

[29] von Eyben F.E., Bauman G., von Eyben R., Rahbar K., Soydal C., Haug A.R., Virgolini I., Kulkarni H., Baum R., Paganelli G. (2020). Optimizing PSMA Radioligand Therapy for Patients with Metastatic Castration-Resistant Prostate Cancer. A Systematic Review and Meta-Analysis. Int. J. Mol. Sci..

[30] Violet J., Jackson P., Ferdinandus J., Sandhu S., Akhurst T., Iravani A., Kong G., Kumar A.R., Thang S.P., Eu P. (2019). Dosimetry of ^177^Lu-PSMA-617 in metastatic castrationresistant prostate cancer: Correlations between pretherapeutic imaging and whole-body tumor dosimetry with treatment outcomes. J. Nucl. Med..

[31] Gunalp B., Emer M., Ozaydin S., Alagoz E., Semra I.N., Ayan A., Alkan S., Mahmudov S., Okuyucu K. (2018). Effectiveness of 177Lu PSMA-617 radioligand therapy in patients with metastatic castration resistant prostate cancer. J. Nucl. Med..

[32] Chandler J.D., Williams E.D., Slavin J.L., Best J.D., Rogers S. (2003). Expression and localization of GLUT1 and GLUT12 in prostate carcinoma. Cancer.

[33] Kostakoglu L., Agress H., Goldsmith S.J. (2003). Clinical role of FDG PET in evaluation of cancer patients. Radiographics.

[34] Liu I.J., Zafar M.B., Lai Y.H., Segall G.M., Terris M.K. (2001). Fluorodeoxyglucose positron emission tomography studies in diagnosis and staging of clinically organ-confined prostate cancer. Urology.

[35] Tateishi U., Morita S., Taguri M., Shizukuishi K., Minamimoto R., Kawaguchi M., Murano T., Terauchi T., Inoue T., Kim E.E. (2010). A meta-analysis of ^18^F-fluoride positron emission tomography for assessment of metastatic bone tumor. Ann. Nucl. Med..

[36] Jadvar H. (2016). Is there utility for FDG PET in prostate cancer. Semin. Nucl. Med..

[37] Lavallée E., Bergeron M., Buteau F.A., Blouin A.C., Duchesnay N., Dujardin T., Tiguert R., Lacombe L., Fradet V., Makao-Nguile M. (2019). Increased prostate cancer glucose metabolism detected by 18F-fluorodeoxyglucose positron emission tomography/computed tomography in localised Gleason 8–10 prostate cancers identifies very high-risk patients for early recurrence and resistance to castration. Eur. Urol. Focus..

[38] Fox J.J., Gavane S.C., Blanc-Autran E., Nehmeh S., Gonen M., Beattie B., Vargas H.A., Schoder H., Humm J.L., Fine S.W. (2018). Positron emission tomog-raphy/computed tomography-based assessments of androgen re-ceptor expression and glycolytic activity as a prognostic biomarker for metastatic castration-resistant prostate cancer. JAMA Oncol..

[39] Hofman M., Emmett L., Sandhu S., Irvani A., Joshua A.M., Goh J.C., Pattison D.A., Tan T.H., Kirkwood I.D., Ng S. (2021). [^177^Lu]Lu-PSMA-617 versus cabazitaxel in patients with metastatic castration-resistant prostate cancer (TheraP): A randomised, open-label, phase 2 trial. Lancet.

[40] Ambrosini V., Kunikowska J., Baudin E., Bodei L., Bouvier C., Capdevila J., Cremonesi M., de Herder W.W., Dromain C., Falconi M. (2021). Consensus on molecular imaging and theranostics in neuroendocrine neoplasms. Eur. J. Cancer.

[41] Emmett L., Willowson K., Violet J., Shin J., Blanksby A., Lee J. (2017). Lutetium 177 PSMA radionuclide therapy for men with prostate cancer: A review of the current literature and discussion of practical aspects of therapy. J. Med. Radiat. Sci..

[42] NuDat 2.8 ^177^Lu. https://www.nndc.bnl.gov/nudat2/decaysearchdirect.jsp?nuc=177LU&unc=nds.

[43] Ruigrok E.A.M., van Vliet N., Dalm S.U., de Blois E., van Gent D.C., Haeck J., de Ridder C., Stuurman D., Konijnenberg M.W., van Weerden W.M. (2021). Extensive preclinical evaluation of lutetium-177-labeled PSMA-specific tracers for prostate cancer radionuclide therapy. Eur. J. Nucl. Med. Mol. Imaging.

[44] de Wit R., de Bono J., Sternberg C.N., Fizazi K., Tombal B., Wulfing C., Kramer G., Eymard J.C., Bamias A., Carles J. (2019). Cabazitaxel versus abiraterone or enzalutamide in metastatic prostate cancer. N. Engl. J. Med..

[45] Santor O., De Bono J.S., Chi K.N., Fizazi K., Herrmann K., Rahbar K., Tagawa S.T., Nordquist L.T., Vaishampayan N., El-Haddad G. (2021). Lutetium-177-PSMA-617 for metastatic castration-resistant prostate cancer. N. Engl. J. Med..

[46] Baum R.P., Kulkarni H.R., Schuchardt C., Singh A., Wirtz M., Wiessalla S., Schottelius M., Mueller D., Klette I., Wester H.J. (2016). ^177^Lu-labeled prostate-specific membrane antigen radioligand therapy of metastatic castration-resistant prostate cancer: Safety and efficacy. J. Nucl. Med..

[47] Heck M.M., Tauber R., Schwaiger S., Retz M., D’Alessandria C., Maurer T., Gafita A., Wester H.J., Gschwend J.E., Weber W.A. (2019). Treatment outcome, toxicity, and predictive factors for radioligand therapy with ^177^Lu-PSMA-I&T in metastatic castration-resistant prostate cancer. Eur. Urol..

[48] Lückerath K., Wei L., Fendler W.P., Evans-Axelsson S., Stuparu A.D., Slavik R., Mona C.E., Calais J., Rettig M., Reiter R.E. (2018). Preclinical evaluation of PSMA expression in response to androgen receptor blockade for theranostics in prostate cancer. EJNMMI Res..

[49] Polkinghorn W.R., Parker J.S., Lee M.X., Kass E.M., Spratt D.E., Iaquinta P.J., Arora V.K., Yen W.F., Cai L., Zheng D. (2013). Androgen receptor signaling regulates DNA repair in prostate cancers. Cancer Discov..

[50] Beer T.M., Armstrong A.J., Rathkopf D.E., Loriot Y., Sternberg C.N., Higano C.S., Iversen P., Bhattacharya S., Carles J., Chowdhury S. (2014). Enzalutamide in metastatic prostate cancer before chemotherapy. N. Engl. J. Med..

[51] Armstrong A.J., Halabi S., Luo J., Nanus D.M., Giannakakou P., Szmulewitz R.Z., Danila D.C., Healy P., Anand M., Rothwell C.J. (2019). Prospective multicenter validation of androgen receptor splice variant 7 and hormone therapy resistance in high-risk castration-resistant prostate cancer: The PROPHECY study. J. Clin. Oncol..

[52] Lord C.J., Ashworth A. (2012). The DNA damage response and cancer therapy. Nature.

[53] Pritchard C.C., Mateo J., Walsh M.F., De Sarkar N., Abiba W., Beltran H., Garofalo A., Gulati R., Carreira S., Eeles R. (2016). Inherited DNA-repair gene mutations in men with metastatic prostate cancer. N. Engl. J. Med..

[54] Robinson D., Van Allen E.M., Wu Y.M., Schultz N., Lonigro R.J., Mosquera J.M., Montgomery B., Taplin M.E., Pritchard C.C., Attard G. (2015). Integrative clinical genomics of advanced prostate. Cancer Cell.

[55] Paschalis A., Sheehan B., Riisnaes R., Rodrigues D.N., Gurel B., Bertan C., Ferreira A., Lambros M.B., Seed G., Yuan W. (2019). Prostate-specific Membrane Antigen Heterogeneity and DNA Repair Defects in Prostate Cancer. Eur. Urol..

[56] de Bono J., Mateo J., Fizazi K., Saad F., Shore N., Sandhu S., Chi K.N., Sartor O., Agarwal N., Olmos D. (2020). Olaparib for metastatic castration resistant prostate cancer. N. Engl. J. Med..

[57] Mateo J., Porta N., Bianchini D., McGovern U., Elliot T., Jones R., Syndikus I., Ralph C., Jain S., Varughese M. (2020). Olaparib in patients with metastatic castration-resistant prostate cancer with DNA repair gene aberrations (TOPARP-B): A multicentre, open-label, randomised, phase 2 trial. Lancet Oncol..

[58] Abida W., Campbell D., Patnaik A., Sautois B., Shapiro J., Vogelzang N.J., Bryce A.H., McDermott R., Ricci F., Rowe J. (2019). 846PD—preliminary results from the TRITON2 study of rucaparib in patients (pts) with DNA damage repair (DDR)-deficient metastatic castration resistant prostate cancer (mCRPC): Updated analyses. Ann. Oncol..

[59] Nonnekens J., van Kranenburg M., Beerens C.E., Suker M., Doukas M., van Eijck C.H., de Jong M., van Gent D.C. (2016). Potentiation of peptide receptor radionuclide therapy by the PARP inhibitor olaparib. Theranostics.

[60] Chalmers A.J. (2004). Poly(ADP-ribose) polymerase-1 and ionizing radiation: Sensor, signaller and therapeutic target. Clin. Oncol. (R Coll Radiol).

[61] Fok J.H.L., Ramos-Montoya A., Vazquez-Chantada M., Wijnhoven P.W.G., Follia V., James N., Farrington P.M., Karmokar A., Willis S.E., Cairns J. (2019). AZD7648 is a potent and selective DNA-PK inhibitor that enhances radiation, chemotherapy and olaparib activity. Nat. Commun..

[62] Riches L.C., Trinidad A.G., Hughes G., Jones G.N., Hughes A.M., Thomason A.G., Gavine P., Cui A., Ling S., Stott J. (2020). Pharmacology of the ATM inhibitor AZD0156: Potentiation of irradiation and olaparib responses preclinically. Mol. Cancer Ther..

[63] Porter L.H., Lawrence M.G., Choo N.K. (2019). PARP Inhibitor and CX-5461 Combination Therapy as a Novel Treatment Strategy for Castrate-Resistant Prostate Cancer. Oncol. Abstr..

[64] Robert C. (2020). A decade of immune-checkpoint inhibitors in cancer therapy. Nat. Commun..

[65] Schumacher T.N., Schreiber R.D. (2015). Neoantigens in cancer immunotherapy. Science.

[66] Graff J.N., Antonarakis E.S., Hoimes C.J., Tagawa S.T., Hwang C., Kilari D., Ten Tije A.J., Omlin A.G., McDermott R.S., Vaishampayan U.N. (2020). Pembrolizumab (pembro) plus enzalutamide (enza) for enza-resistant metastatic castration resistant prostate cancer (mCRPC): KEYNOTE-199 cohorts 4–5. J. Clin. Oncol..

[67] Kwon E.D., Drake C.G., Scher H.I., Fizazi K., Bossi A., Van den Eertwegh A.J., Krainer M., Houede N., Santos R., Mahammedi H. (2014). Ipilimumab versus placebo after radiotherapy in patients with metastatic castration-resistant prostate cancer that had progressed after docetaxel chemotherapy (CA184–043): A multicentre, randomised, double-blind, phase 3 trial. Lancet Oncol..

[68] Demaria S., Ng B., Devitt M.L., Babb J.S., Kawashima N., Liebes L., Formenti S.C. (2004). Ionizing radiation inhibition of distant untreated tumors (abscopal effect) is immune mediated. Int. J. Radiat. Oncol. Biol. Phys..

[69] O’Dwyer E., Bodei L., Morris M.J. (2021). The role of theranostics in prostate cancer. The Role of Theranostics in Prostate Cancer. Semin. Radiat. Oncol..

[70] Zechmann C.M., Afshar-Oromieh A., Armor T., Stubbs J.B., Mier W., Hadaschik B., Joyal J., Kopka K., Debus J., Babich J.W. (2014). Radiation dosimetry and first therapy results with a ^124^I/^131^I-labeled small molecule (MIP-1095) targeting PSMA for prostate cancer therapy. Eur. J. Nucl. Med. Mol. Imaging.

[71] Afshar-Oromieh A., Haberkorn U., Zechmann C., Armor T., Mier W., Spohn F., Debus N., Holland-Letz T., Babich J., Kratochwil C. (2017). Repeated PSMA-targeting radioligand therapy of metastatic prostate cancer with ^131^I-MIP-1095. Eur. J. Nucl. Med. Mol. Imaging.

[72] Muzio V., Ravasi L., Sacchetti L., Fugazza L., Bacot S., Debiossat M., Ahmadi M., Montemagno C., Ghezzi C., Broisat A. Biodistribution of PSMA-R2 in mice bearing prostate cancer. Proceedings of the EANM 19.

[73] Kassis A.I., Adelstein S.J. (2005). Radiobiologic principles in radionuclide therapy. J. Nucl. Med..

[74] Pouget J.P., Navarro-Teulon I., Bardies M., Chouin N., Cartron G., Pelegrin A., Azria D. (2011). Clinical radioimmunotherapy—The role of radiobiology. Nat. Rev. Clin. Oncol..

[75] Dekempeneer Y., Keyaerts M., Krasniqi A., Puttemans J., Muyldermans S., Lahoutte T., D’huyvetter M., Devoogdt N. (2016). Targeted alpha therapy using short-lived alpha-particles and the promise of nanobodies as targeting vehicle. Expert Opin. Biol. Ther..

[76] Kratochwil C., Bruchertseifer F., Giesel F.L., Weis M., Verburg F.A., Mottaghy F., Kopka K., Apostolidis C., Haberkorn U., Morgenstern A. (2016). 225Ac-PSMA-617 for PSMA-targeted alpha-radiation therapy of metastatic castration resistant prostate cancer. J. Nucl. Med..

[77] Kratochwil C., Bruchertseifer F., Rathke H., Bronzel M., Apstolidis C., Weichert W., Haberkorn U., Giesel F.L., Morgenstern A. (2017). Targeted alpha therapy of mCRPC with ^225^Actinium-PSMA-617: Dosimetry estimate and empirical dose finding. J. Nucl. Med..

[78] Feuerecker B., Tauber R., Knorr K., Heck M., Beheshti A., Seidl C., Bruchertseifer F., Pickhard A., Gafita A., Kratochwil C. (2021). Activity and Adverse Events of Actinium-225-PSMA-617 in Advanced Metastatic Castration-resistant Prostate Cancer after Failure of Lutetium-177-PSMA. Eur. Urol..

[79] Sathekge M.M., Bruchertseifer F., Lawal I.O., Vorster M., Knoesen O., Lengana T., Boshomane T.G., Mokoala K.K., Morgenstern A. (2019). Treatment of brain metastases of castration-resistant prostate cancer with ^225^Ac-PSMA-617. Eur. J. Nucl. Med. Mol. Imaging.

[80] McDevitt M.R., Barendswaard E., Ma D., Lai L., Curcio M.J., Sgouros G., Ballangrud A.M., Yang W.H., Finn R.D., Pellegrini V. (2000). An alpha-particle emitting antibody ([^213^Bi]J591) for radioimmunotherapy of prostate cancer. Cancer Res..

[81] Li Y., Tian Z., Rizvi S.M., Bander N.H., Allen B.J. (2002). In vitro and preclinical targeted alpha therapy of human prostate cancer with Bi-213 labeled J591 antibody against the prostate specific membrane antigen. Prostate Cancer Prostatic Dis..

[82] Sathekge M., Knoesen O., Meckel M., Modiselle M., Vorster M., Marx S. (2017). ^213^Bi-PSMA-617 targeted alpha-radionuclide therapy in metastatic castration-resistant prostate cancer. Eur. J. Nucl. Med. Mol. Imaging.

[83] Kratochwil C., Schmidt K., Afshar-Oromieh A., Bruchertseifer F., Rathke H., Morgenstern A., Haberkorn U., Giesel F.L. (2018). Targeted alpha therapy of mCRPC: Dosimetry estimate of ^213^Bismuth-PSMA-617. Eur. J. Nucl. Med. Mol. Imaging.

[84] Liu H., Moy P., Kim S., Xia Y., Rajasekaran A., Navarro V., Knudsen B., Bander N.H. (1997). Monoclonal antibodies to the extracellular domain of prostate-specific membrane antigen also react with tumor vascular endothelium. Cancer Res..

[85] Bander N.H., Milowsky M.I., Nanus D.M., Kostakoglu L., Vallabhajosula S., Goldsmith S.J. (2005). Phase I trial of ^177^lutetium-labeled J591, a monoclonal antibody to prostate-specific membrane antigen, in patients with androgen-independent prostate cancer. J. Clin. Oncol..

[86] Tagawa S.T., Milowsky M.I., Morris M., Vallabhajosula S., Christos P., Akhtar N.H., Osborne J., Goldsmith S.J., Larson S., Taskar N.P. (2013). Phase II study of Lutetium-177-labeled anti-prostate-specific membrane antigen monoclonal antibody J591 for metastatic castration-resistant prostate cancer. Clin. Cancer Res..

[87] Tagawa S.T., Vallabhajosula S., Christos P.J., Jhanwar Y.S., Batra J.S., Lam L., Osborne J., Beltran H., Molina A.M., Goldsmith S.J. (2019). Phase 1/2 study of fractionated dose lutetium-177-labeled anti-prostate-specific membrane antigen monoclonal antibody J591 (^177^Lu-J591) for metastatic castration-resistant prostate cancer. Cancer.

[88] Tagawa S.T., Osborne J., Fernandez E., Thomas C., Niaz M.J., Ciriaco A., Vallabhajosula S., Vlachostergios P.J., Molina A.M., Sternberg C.N. (2020). Phase I dose-escalation study of PSMA-targeted alpha emitter ^225^Ac-J591 in men with metastatic castration-resistant prostate cancer (mCRPC). J. Clin. Oncol..

[89] Hammer S., Hagemann U.B., Zitzmann-Kolbe S., Larsen A., Ellingsen C., Geraudie S., Grant D., Indrevoll B., Smeets R., von Ahsen O. (2020). Preclinical Efficacy of a PSMA-Targeted Thorium-227 Conjugate (PSMA-TTC), a Targeted Alpha Therapy for Prostate Cancer. Clin. Cancer Res..

[90] Goebeler M.E., Knop S., Viardot A., Kufer P., Topp M.S., Einsele H., Noppeney R., Hess G., Kallert S., Mackensen A. (2016). Bispecific T-cell engager (BiTE) antibody construct blinatumomab for the treatment of patients with relapsed/refractory non-Hodgkin lymphoma: Final results from a phase I study. J. Clin. Oncol..

[91] Kantarjian H.M., Stein A., Gökbuget N., Fielding A.K., Schuh A.C., Ribera J.-M., Wei A., Dombret H., Foà R., Bassan R. (2017). Blinatumomab versus chemotherapy for advanced acute lymphoblastic leukemia. N. Engl. J. Med..

[92] Bargou R., Leo E., Zugmaier G., Klinger M., Goebeler M., Knop S., Noppeney R., Viardot A., Hess G., Schuler M. (2008). Tumor regression in cancer patients by very low doses of a T cell-engaging antibody. Science.

[93] Hummel H.D., Kufer P., Grüllich C., Deschler-Baier B., Chatterjee M., Goebeler M.E., Miller K., De Santis M., Loidl W.C., Buck A. (2020). Phase I study of pasotuxizumab (AMG 212/BAY 2010112), a PSMA-targeting BiTE (bispecific T-cell engager) immune therapy for metastatic castration-resistant prostate cancer (mCRPC). J. Clin. Oncol..

